# Supplementary material for: Characterising the epidemic spread of influenza A/H3N2 within a city through phylogenetics
Source: PLoS Pathog. 2020 Nov 19;16(11):e1008984. doi: 10.1371/journal.ppat.1008984 (PMC7676729; doi:10.1371/journal.ppat.1008984)
Supplement: S1 Text — (PDF) [file ppat.1008984.s001.pdf]

Overall, 857 cases were PCR positive for Influenza A (845) or Influenza B (12) Overall, the dataset included sequencing runs from 845 unique isolates of influenza A samples. For some of these isolated, sequencing was performed more than once. In this case, the isolate that fulfilled the quality criteria detailed below better was used. These isolates, in some cases, were isolated from the same patient, but at different time points. Bases were called to be neutral if the coverage for a site was less than 100. If a segment of a sequence had more than 20% neutral bases on more than 5 segments, the sequence was not used for further analysis. If the HA segment was not part of the 3 segments that were had less than 20% neutral bases, the sequence was discarded as well. Using the same criteria but requiring at least 4 segments, would have resulted in 736 sequences to be used. For 5 segments, 728 would have passed, 719 for 6 segments, 713 for 7 and 664 for 8. This quality criteria was most likely violated by the NA segment, explaining the drop of isolates that would have been used when requiring all 8 segments instead of 7. For patients with more than 1 isolate that passed the quality control, the sequence that was isolated earlier was used.
