# Supplementary figures and images for: Characterising the epidemic spread of influenza A/H3N2 within a city through phylogenetics
Source: PLoS Pathog. 2020 Nov 19;16(11):e1008984. doi: 10.1371/journal.ppat.1008984 (PMC7676729; doi:10.1371/journal.ppat.1008984)

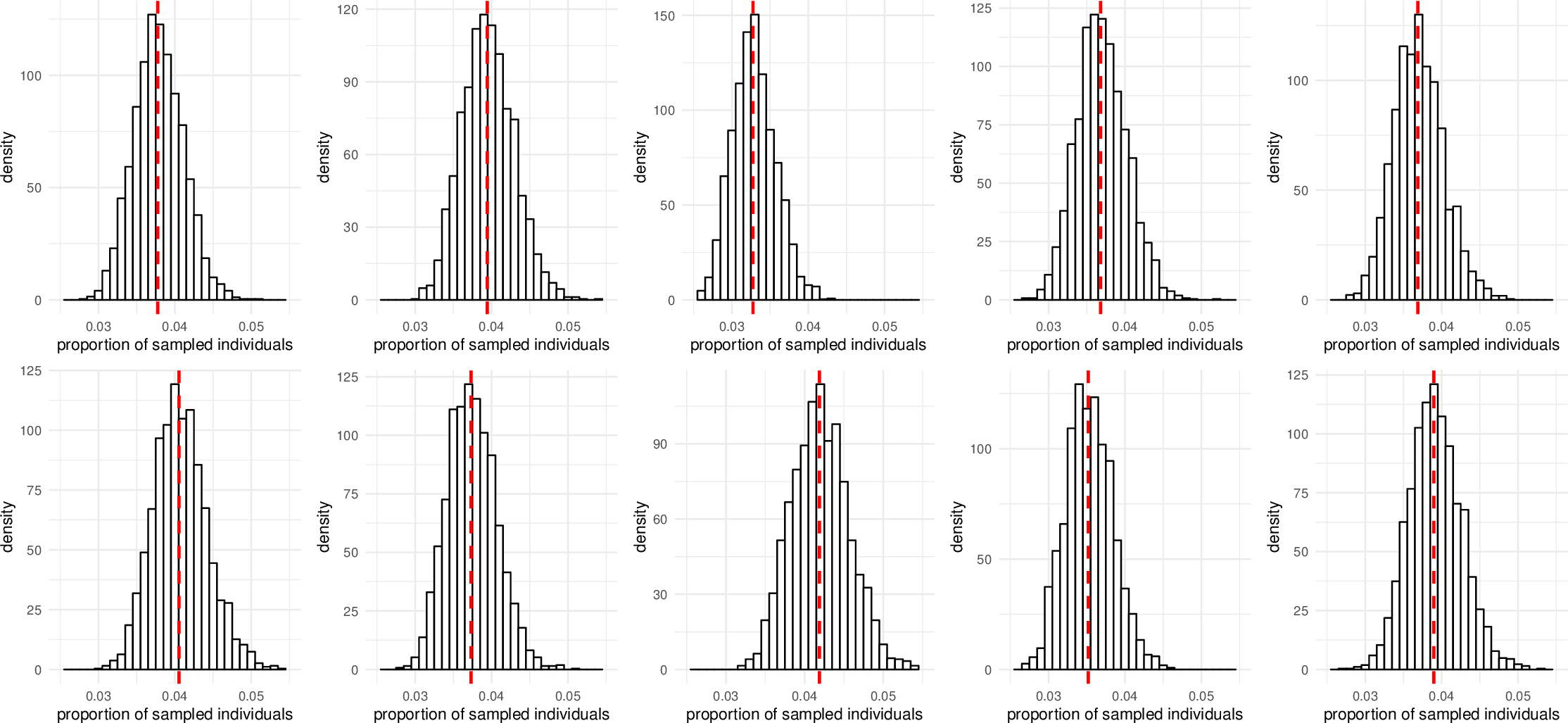

Supplement: S1 Fig — Each histogram shows an inferred sampling proportion based on a classification of sequences into local clusters. Since these classifications are dependent on which iteration of the MCMC was used for the classification into local clusters, we repeated the analysis using 10 different random iterations. Each subplot shows the estimated sampling proportion when using one of these classifications. The dotted red line shows the median estimate of the sampling proportion over all iterations. (TIF) [file ppat.1008984.s002.tif]

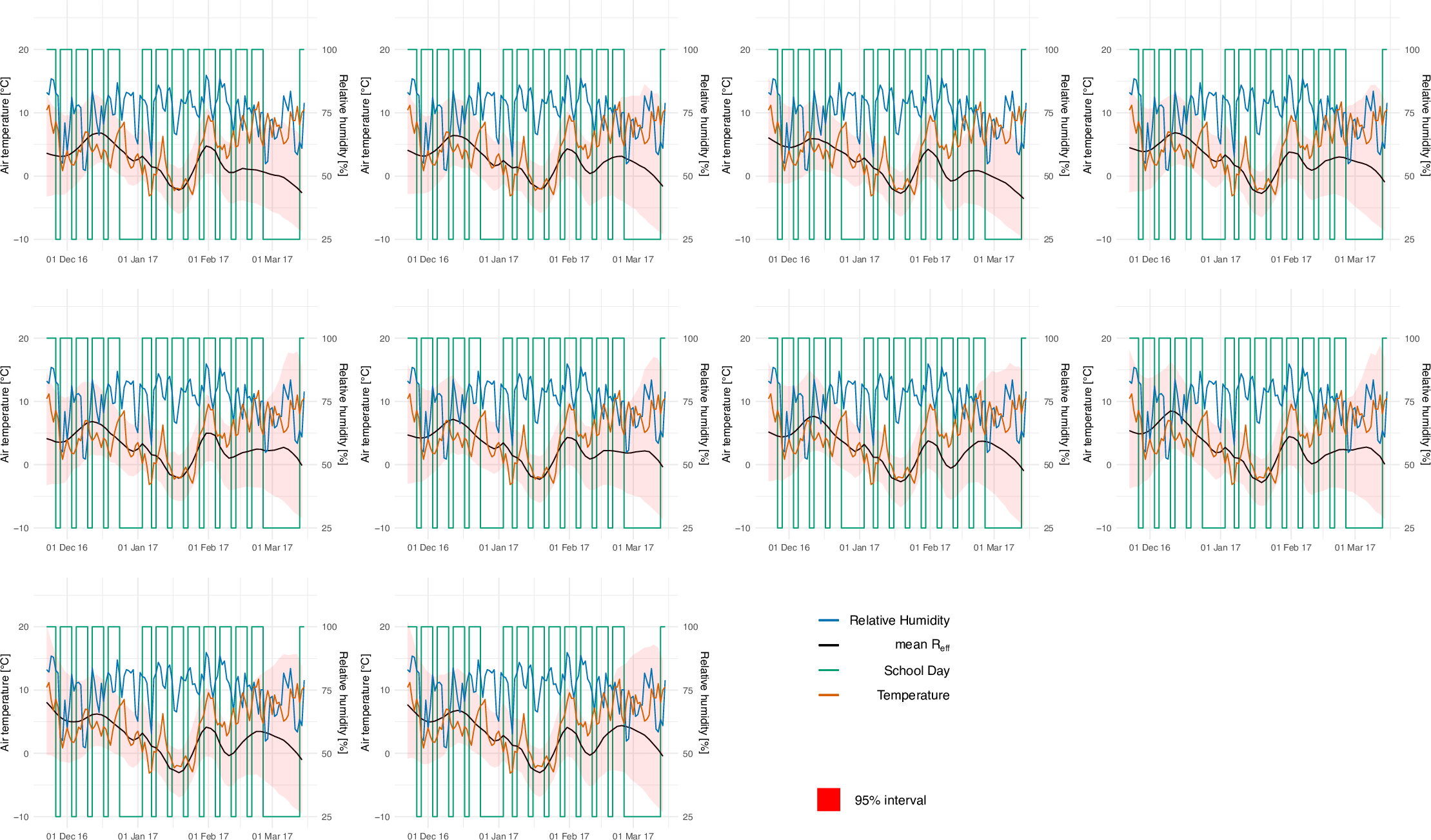

Supplement: S2 Fig — Each subplot shows the inferred effective reproduction number when using a different iteration of the MCMC for the assignment of Basel sequences into local clusters. (TIF) [file ppat.1008984.s003.tif]

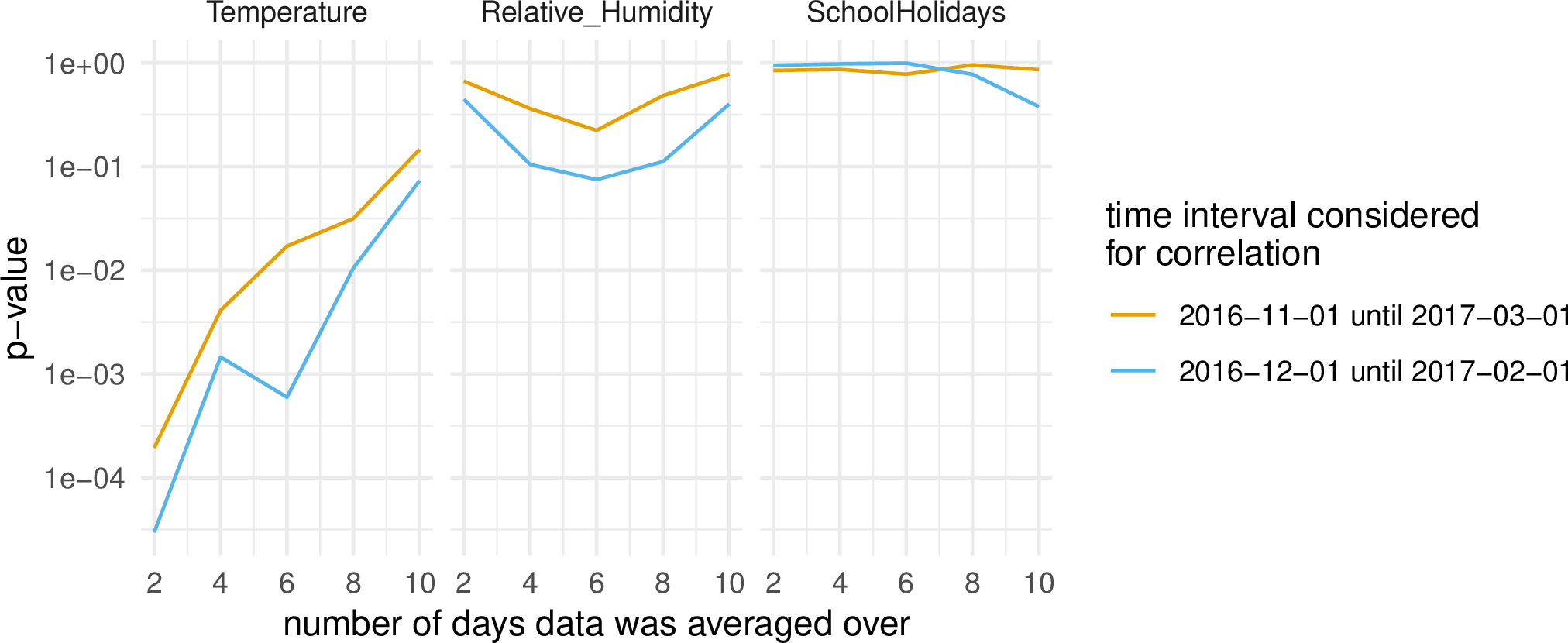

Supplement: S3 Fig — Here we show the estimated p-values for the correlation between the effective reproduction number and temperature, relative humidity and school days estimated when the data was averaged over different number of days (x-axis). The estimate p-values are shown for two different time intervals (in different colors). For the orange line, estimates for 1 November 2016 until 1 March 2017 were used and for the blue line, estimated from December until February were used. These plots were generated using the effective reproduction number averaged over 10 different classifications of sequences into local clusters. The equivalent plots for generated using the effective reproduction number estimates of each individual subset is shown in S5 Fig. (TIF) [file ppat.1008984.s004.tif]

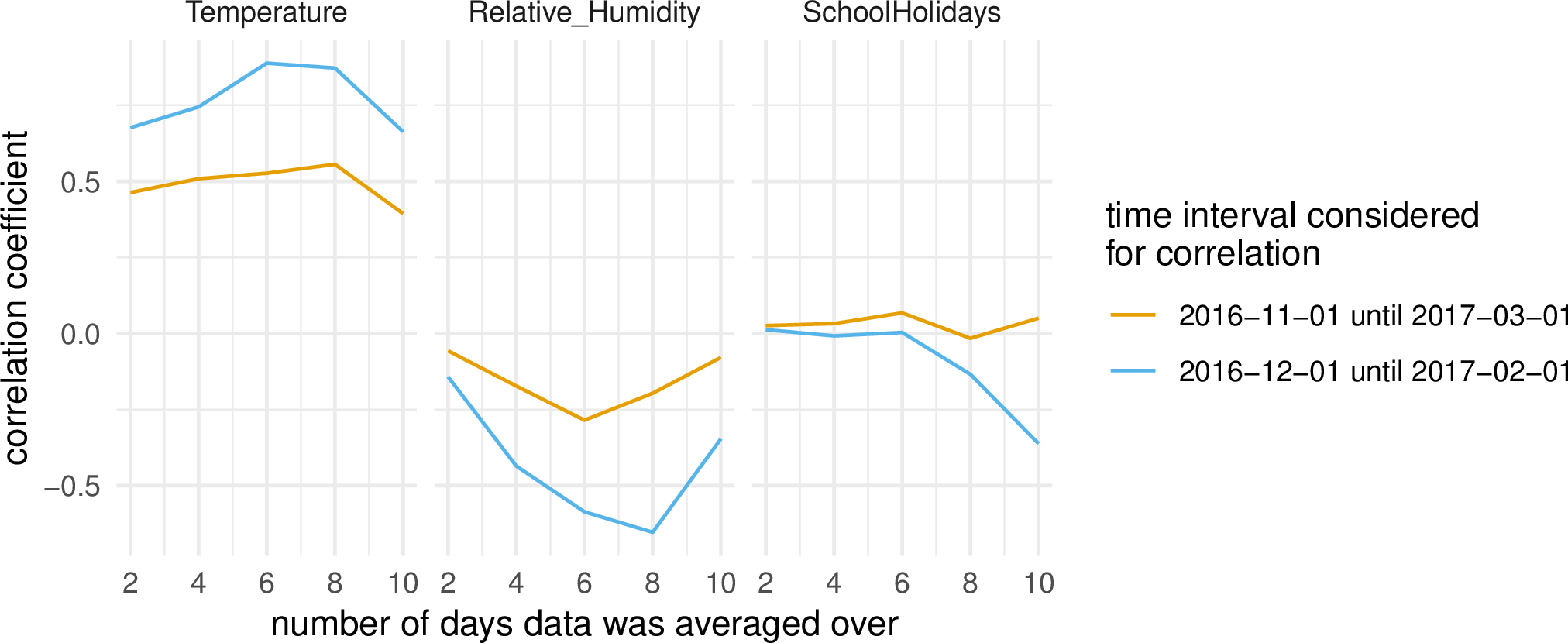

Supplement: S4 Fig — Here we show the estimated correlation coefficients for the correlation between the effective reproduction number and temperature, relative humidity and school days estimated when the data was averaged over different number of days (x-axis). The estimate p-values are shown for two different time intervals (in different colors). For the orange line, estimates for 1 November 2016 until 1 March 2017 were used and for the blue line, estimated from December until February were used. These plots were generated using the effective reproduction number averaged over 10 different classifications of sequences into local clusters. The equivalent plots for generated using the effective reproduction number estimates of each individual subset is shown in S6 Fig. (TIF) [file ppat.1008984.s005.tif]

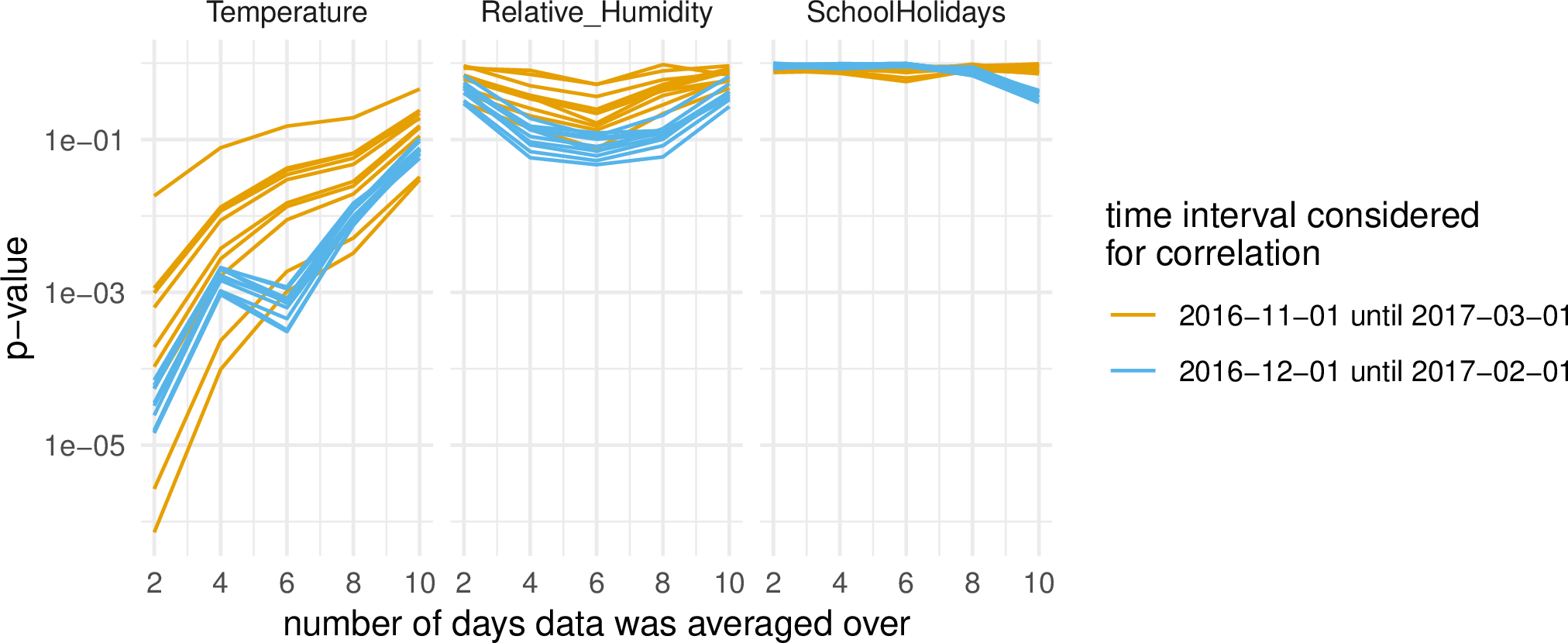

Supplement: S5 Fig — Here we show the estimated p-values for the correlation between the effective reproduction number and temperature, relative humidity and school days estimated when the data was averaged over different number of days (x-axis). The estimate p-values are shown for two different time intervals (in different colors). For the orange line, estimates for 1 November 2016 until 1 March 2017 were used and for the blue line, estimated from December until February were used. The different lines of the same color show the p-values estimates using the effective reproduction number estimates of individual classifications of sequences into local clusters. (TIF) [file ppat.1008984.s006.tif]

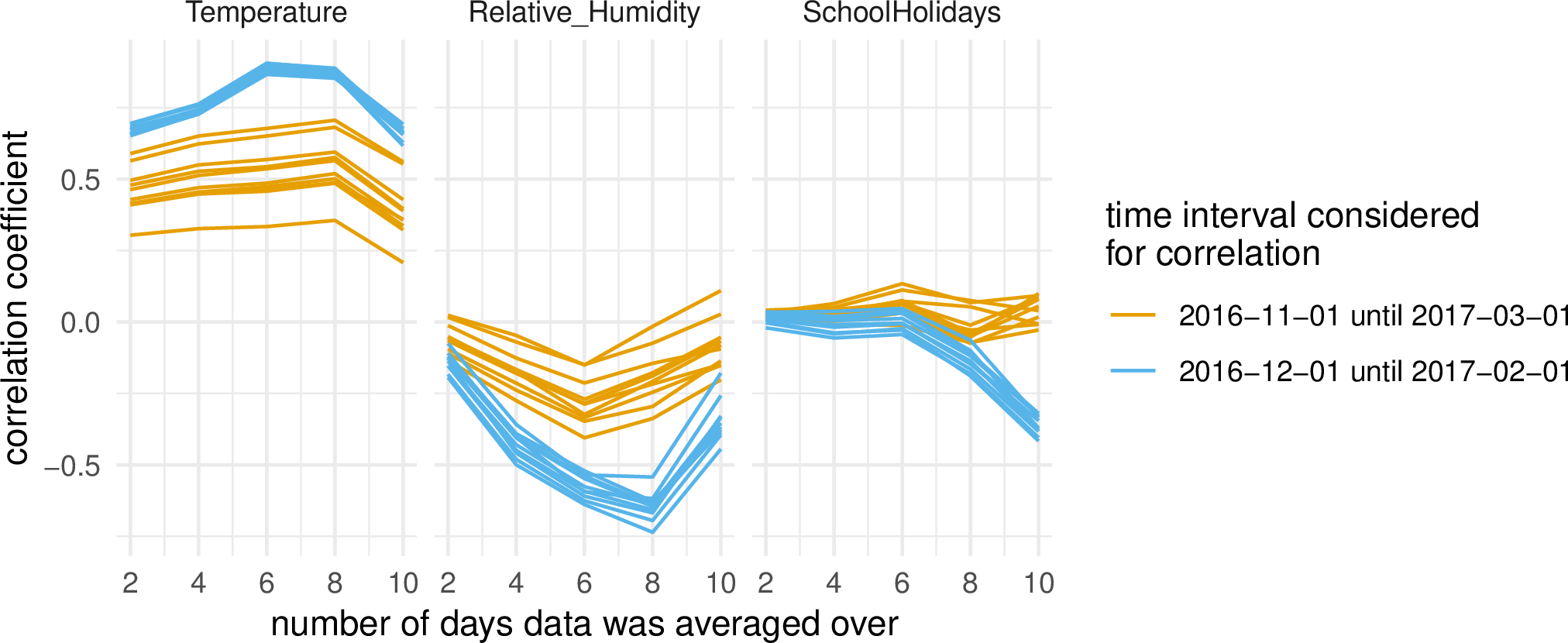

Supplement: S6 Fig — Here we show the estimated correlation coefficients for the correlation between the effective reproduction number and temperature, relative humidity and school days estimated when the data was averaged over different number of days (x-axis). The estimate p-values are shown for two different time intervals (in different colors). For the orange line, estimates for 1 November 2016 until 1 March 2017 were used and for the blue line, estimated from December until February were used. The different lines of the same color show the p-values estimates using the effective reproduction number estimates of individual classifications of sequences into local clusters. (TIF) [file ppat.1008984.s007.tif]

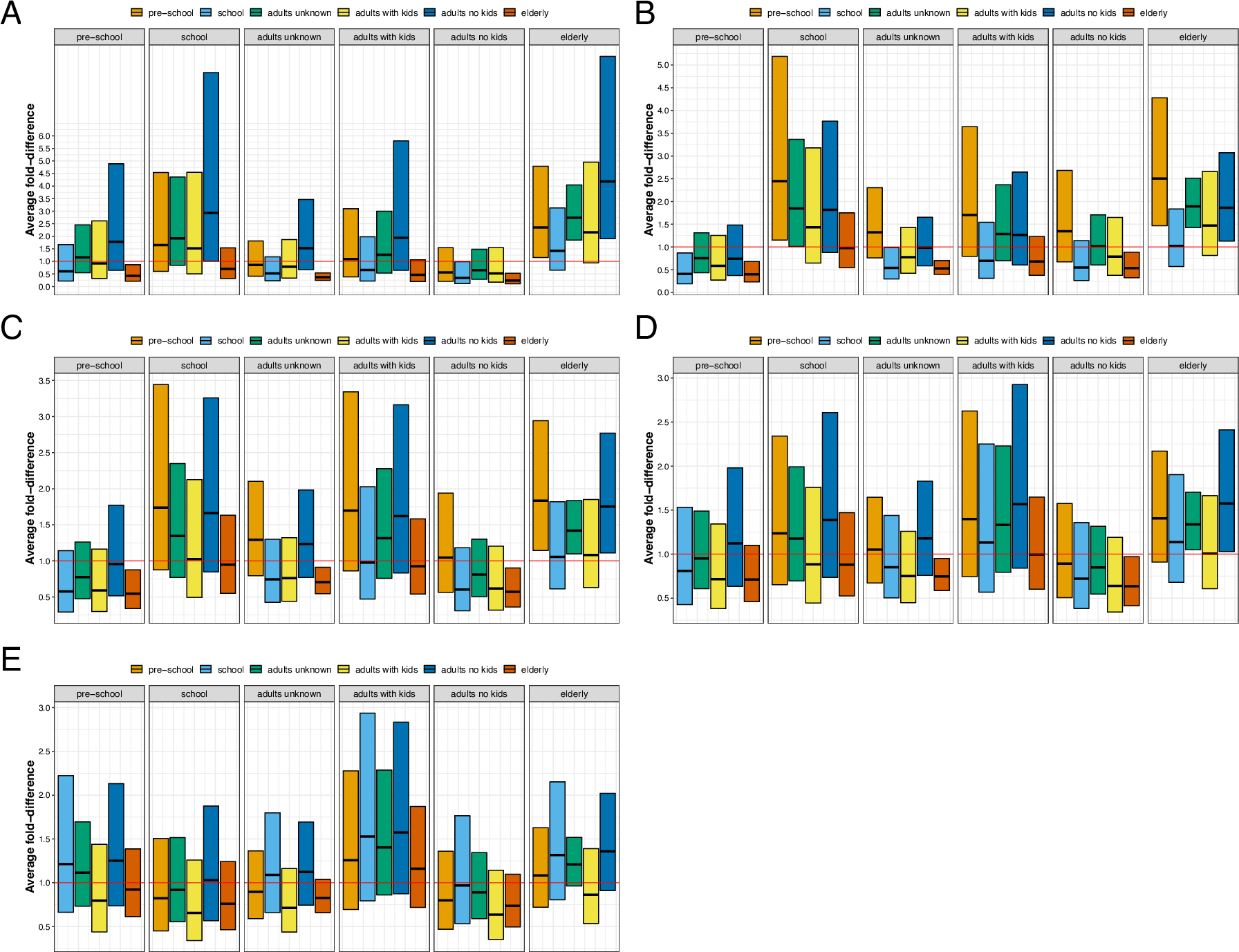

Supplement: S7 Fig — Plots are analogue to Fig 2c, but for different thresholds: 0.05 years in plot A, 0.1 years in plot B (analogue to Fig 2c), 0.15 years in plot C, 0.2 years in plot D and 0.3 years in plot E. (TIF) [file ppat.1008984.s008.tif]

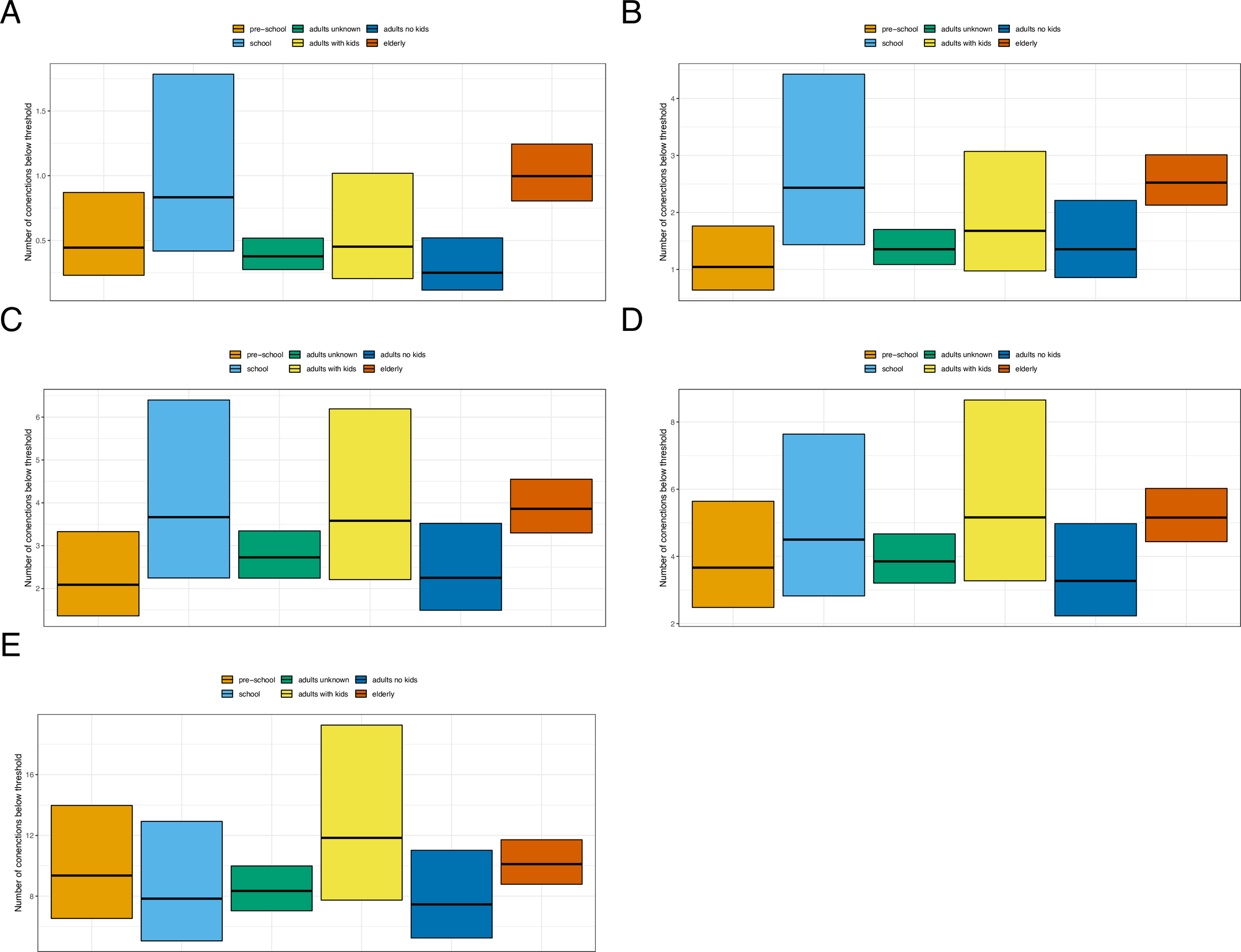

Supplement: S8 Fig — Each subplot shows the average number of connected individuals a patient from the group shown by the color is connected to. Upper and lower bounds correspond to 95% confidence intervals around the average. We consider two patients to be connected if the pairwise phylogenetic distance between the influenza viruses sequenced from them is below a certain threshold. These thresholds are 0.05 years in plot A, 0.1 years in plot B, 0.15 years in plot C, 0.2 years in plot D and 0.3 years in plot E. (TIF) [file ppat.1008984.s009.tif]

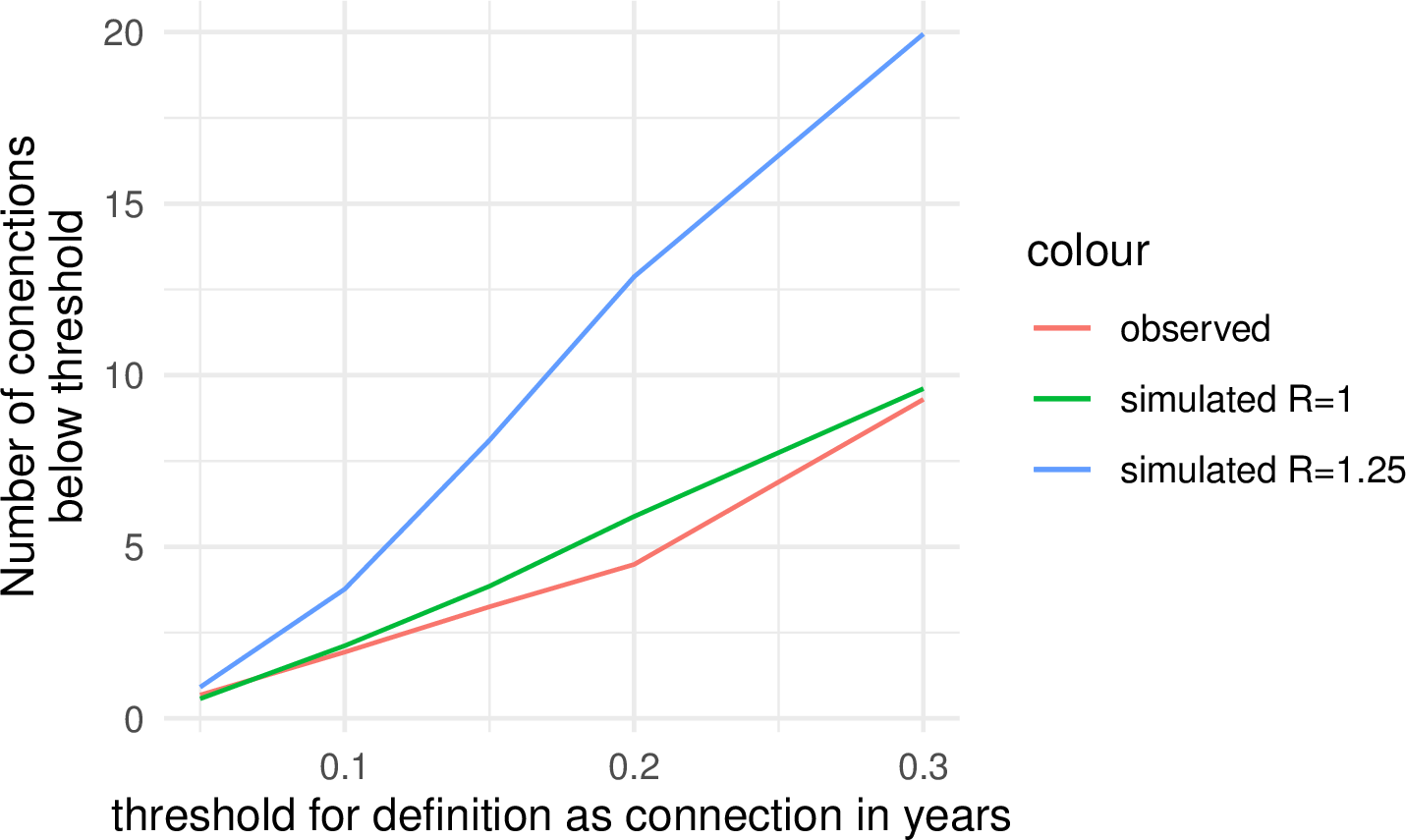

Supplement: S9 Fig — Here, we compare the average number of connections (y-axis) of an individual patient to other patients for different threshold (x-axis) between what we observe empirically and what we observe in simulations. We ran the simulations using either a reproduction number of 1 or 1.25, a becoming uninfectious rate of 0.25 per day and a sampling proportion of 4%. (TIF) [file ppat.1008984.s010.tif]

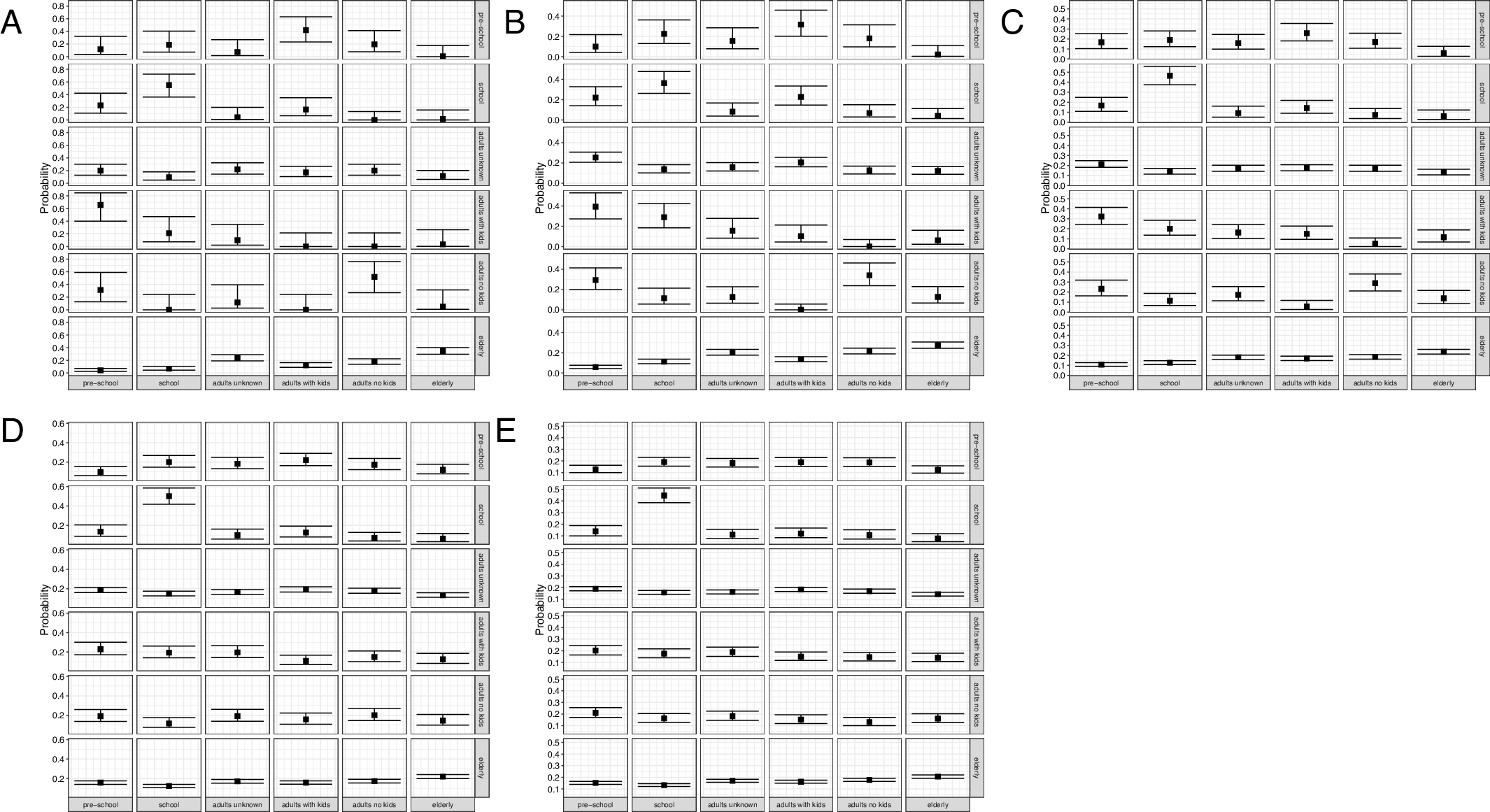

Supplement: S10 Fig — Plots are analogue to Fig 3a, but for different thresholds: 0.05 years in plot A, 0.1 years in plot B, 0.15 years in plot C, 0.2 years in plot D and 0.3 years in plot E. (TIF) [file ppat.1008984.s011.tif]

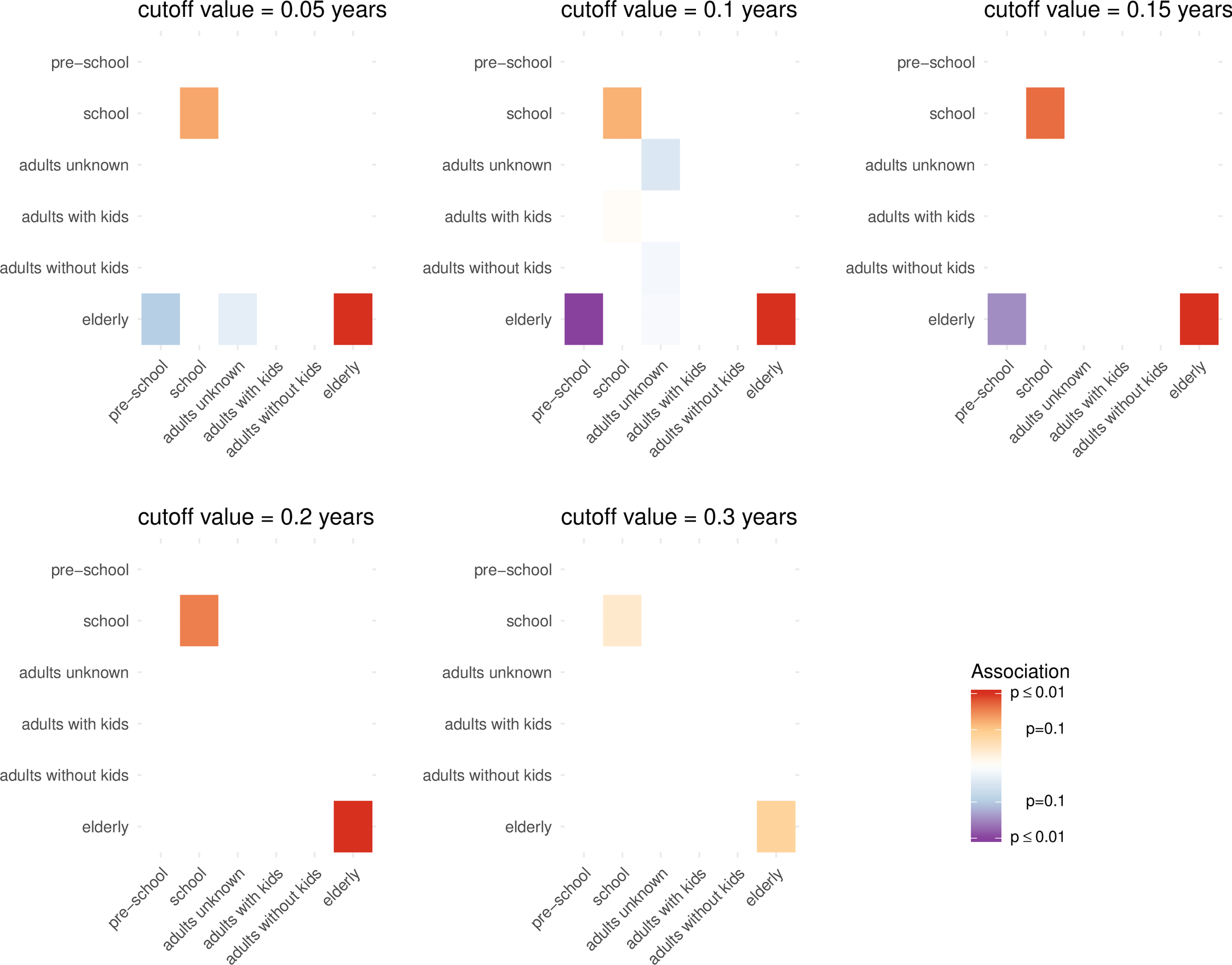

Supplement: S11 Fig — Plots are analogue to Fig 3b, but for different thresholds. The thresholds in years are given on top of each subfigure. Unless very large thresholds are used, the elderly are estimated to be positively associated with other members from the same group. School aged children are associated with other school aged children except for very low thresholds where the number of pairs is very low. Elderly being negatively associated with pre-school aged children shows up only at lower thresholds. (TIF) [file ppat.1008984.s012.tif]
